# Prediction of Treatment Outcome in Patients with Obsessive-Compulsive Disorder with Low-Resolution Brain Electromagnetic Tomography: A Prospective EEG Study

**DOI:** 10.3389/fpsyg.2015.01993

**Published:** 2016-01-22

**Authors:** Daniela Krause, Malte Folkerts, Susanne Karch, Daniel Keeser, Agnieszka I. Chrobok, Michael Zaudig, Ulrich Hegerl, Georg Juckel, Oliver Pogarell

**Affiliations:** ^1^Department of Neurophysiology and Functional Neuroimaging, Ludwig Maximilians University MunichMunich, Germany; ^2^Psychosomatic ClinicWindach, Germany; ^3^Department of Psychiatry and Psychotherapy, University Hospital LeipzigLeipzig, Germany; ^4^Department of Psychiatry, Psychotherapy and Preventive Medicine, Ruhr-University BochumBochum, Germany

**Keywords:** treatment prediction, obsessive–compulsive disorder, LORETA, anterior cingulate cortex, EEG

## Abstract

The issue of predicting treatment response and identifying, in advance, which patient will profit from treating obsessive-compulsive disorder (OCD) seems to be an elusive goal. This prospective study investigated brain electric activity [using Low-Resolution Brain Electromagnetic Tomography (LORETA)] for the purpose of predicting response to treatment. Forty-one unmedicated patients with a DSM-IV diagnosis of OCD were included. A resting 32-channel EEG was obtained from each participant before and after 10 weeks of standardized treatment with sertraline and behavioral therapy. LORETA was used to localize the sources of brain electrical activity. At week 10, patients were divided into responders and non-responders (according to a reduction of symptom severity >50% on the Y-BOCS). LORETA analysis revealed that at baseline responders showed compared to non-responders a significantly lower brain electric activity within the beta 1 (*t* = 2.86, *p* < 0.05), 2 (*t* = 2.81, *p* < 0.05), and 3 (*t* = 2.76, *p* < 0.05) frequency bands and ROI analysis confirmed a reduced activity in alpha 2 (*t* = 2.06, *p* < 0.05) in the anterior cingulate cortex (ACC). When baseline LORETA data were compared to follow-up data, the analysis showed in the responder group a significantly lower brain electrical resting activity in the beta 1 (*t* = 3.17. *p* < 0.05) and beta 3 (*t* = 3.11. *p* < 0.05) frequency bands and equally for the ROI analysis of the orbitofrontal cortex (OFC) in the alpha 2 (*t* = 2.15. *p* < 0.05) frequency band. In the group of non-responders the opposite results were found. In addition, a positive correlation between frequency alpha 2 (rho = 0.40, *p* = 0.010), beta 3 (rho = 0.42, *p* = 0.006), delta (rho = 0.33, *p* = 0.038), theta (rho = 0.34, *p* = 0.031), alpha 1 (rho = 0.38, *p* = 0.015), and beta1 (rho = 0.34, *p* = 0.028) of the OFC and the bands delta (rho = 0.33, *p* = 0.035), alpha 1 (rho = 0.36, *p* = 0.019), alpha 2 (rho = 0.34, *p* = 0.031), and beta 3 (rho = 0.38, *p* = 0.015) of the ACC with a reduction of the Y-BOCS scores was identified. Our results suggest that measuring brain activity with LORETA could be an efficient and applicable technique to prospectively identify treatment responders in OCD.

## Introduction

Obsessive-compulsive disorder (OCD) has a life-time prevalence of 0.8–2% and is characterized by the presence of obsessions and/or compulsions (Baldwin et al., 2005). Patients suffering from this disorder are best treated with a combination therapy consisting of a selective serotonin reuptake inhibitor (SSRI) and cognitive behavioral therapy (CBT), using exposure and response prevention techniques. However, not all patients profit from this treatment. Up to 30% of OCD patients treated with this combination therapy show no or poor improvement after this standard treatment (Ferguson, 2001). Differences of treatment response suggest the existence of biological differences among groups of OCD patients. Therefore, it would be desirable to distinguish responders and non-responders before initiation of the treatment course. The non-responder group could possibly profit from early on augmentation treatment strategies and a closer clinical monitoring. To date some studies have tried to identify helpful tool for predicting therapy response in OCD patients. One genome-wide association study indicated suggestive roles of genes in the glutamatergic neurotransmission and the serotonergic system as genetic predictors of treatment response in the OCD patient cohort (Qin et al., 2015). Also, longer duration of illness was found to be a significant predictor of remission (Eisen et al., 2013). One PET imaging study showed that a reduced regional cerebral blood flow in the orbitofrontal cortex (OFC) and higher values in the posterior cingulate cortex predicted better treatment response to fluvoxamine (Rauch et al., 2002). However, studies investigating prognostic biomarkers that are widely available and can be measured before treatment are rare. One pilot study examined the possibility of predicting treatment response in OCD patients with low-resolution electromagnetic tomography and investigated therefore 17 drug-free OCD patients. Over the course of a 12 week treatment with antidepressants (clomipramine, fluoxetine, sertraline, paroxetine, imipramine, nortiptyline, venlafaxine, and mirtazapine), LORETA values were calculated twice. The 17 patients were classified as responders and non-responders according to a reduction of at least 35% in the YBOCS score. The authors found that responders exhibited significantly lower activities in the beta band in the rostral anterior cingulate and the medial frontal gyrus (Fontenelle et al., 2006). Though the results have to been seen as preliminary as the sample size was rather small and OCD patients did not have a standardized treatment. In addition, functional imaging studies have revealed an abnormally increased activity in the OFC, the basal ganglia and the anterior cingulate cortex (ACC) in patients with OCD (Alptekin et al., 2001; Lacerda et al., 2003), whereas successful pharmacological or psychotherapeutic treatment of OCD patients was associated with a reduction of aberrant activity in the OFC area (Aouizerate et al., 2004).

The present study is based on the previously above mentioned results mainly the findings from Fontenelle et al. and aimed to further investigate whether Low-Resolution Brain Electromagnetic Tomography (LORETA; Pascual-Marqui et al., 1994), a three-dimensional EEG source localization technique, could be a helpful tool to identify whether OCD patients that show a clinical improvement over the course of treatment compared to those patients who do not respond show differences in LORETA values before and/or after standardized treatment. In addition, the correlation between clinical treatment effects and the electromagnetic tomography data was evaluated.

## Materials and methods

### Subjects

In total, forty-one patients with OCD (18 women, 23 men; average age 34.5 years, SD: 9.8; mean duration of illness: 12.8 years, SD 9.3) participated in the study. All patients were diagnosed according to DSM-IV and ICD-10. Symptom severity was assessed using the Yale–Brown Obsessive–Compulsive Scale (Y-BOCS; Goodman et al., 1989), where a score of at least 18 was required for inclusion. In addition, the Maudsley Obsessive–Compulsive Scale (MOCI; Hodgson and Rachman, 1977) for the assessment of OCD symptoms and the Hamilton Depression Scale (HAMD-17; Hamilton, 1960) as well as the Beck Depression Inventory (BDI; Beck et al., 1961) for the assessment of comorbid depressive symptomatology were applied. Moreover, the State-Trait Anxiety Inventory (STAI; Laux et al., 1981) was used in order to assess state and trait anxiety in patients. Subjects had to be free of comorbid psychiatric, neurological or severe somatic diagnoses. All patients were either unmedicated or had undergone a wash-out period of at least 2 weeks. The study was reviewed and approved by the local ethics committee of the Ludwig–Maximilians University and was carried out in accordance with the Declaration of Helsinki. All subjects gave written informed consent for participation in this study, after design and procedures had been fully explained.

### Study design

A neurophysiological baseline recording of resting EEG was performed in all patients. After this initial examination, patients received for 10 weeks a combination therapy consisting of an antidepressant and psychotherapy. All patients received the selective serotonin reuptake inhibitor (SSRI) sertraline. The initial dose was 50 mg/d for 4 weeks unchanged. Patients that showed a reduction of <10% in the Y-BOCS score after 4 weeks on treatment received a dose augmentation to 100 mg/d sertraline and after 7 weeks an increase to 150 mg/d was possible. In addition, all patients received semi-structured CBT (with flooding, family and couple therapy) as an inpatient at the Psychosomatic clinic Roseneck. At the end of the 10 week treatment phase, a second EEG was performed in all study participants. Treatment response was defined as a 50% or more decrease in the Y-BOCS score after 10 weeks treatment with sertraline and behavior therapy. The 50% reduction criterion was chosen according to reports by Tolin et al. (2005) who demonstrated that a Y-BOCS reduction of 40–50% may be optimal for predicting mild illness or better at posttreatment (CGI 3), and Lewin et al. (2011) who showed that a 35% Y-BOCS reduction may be sufficient for clinical use, but for research 45% or even 55% may be more efficient.

### EEG data acquisition and loreta analysis

For EEG recording, patients were seated in a sound-attenuated, electrically shielded room in a reclining chair with eyes closed (wakeful-resting condition). Electrodes were placed via electrocaps (Electro-Cap International, Inc., Eaton, USA) according to the 10/20 system with Cz as reference and Fpz as ground electrode. Additional electrodes (above the left eye and at the left ocular canthis) were used to record the electrooculogram (EOG) simultaneously. Electrode skin impedance was <5kΩ throughout the session. Data were collected with a sampling rate of 250 Hz and an analogous bandpass filter (0.16–50 Hz). All EEG recordings lasted 40 min. Before analysis, artifact detection was visually performed and additionally, the EEG was analyzed four times independently by two experienced neurophysiologists. When artifact detection was finished, segments with 2048 ms (512 data points) were chosen for further processing. A priori it was determined that only subjects with at least 40 artifact-free epochs would be included. The analyses of the frequency and localization were performed with the LORETA (low resolution brain electromagnetic tomography) software package (Pascual-Marqui et al., 1999). The following (standard definitions) frequency bands of LORETA cross spectrum analysis were identified for: delta (1.5–6.0 Hz), theta (6.5–8.0 Hz, alpha1 (8.5–10.0 Hz), alpha2 (10.5–12.0 Hz), beta1 (12.5–18.0 Hz), beta2 (18.5–21.0 Hz), and beta3 (21.5–30.0 Hz). The method LORETA assumes that the smoothest of all activity distributions is most plausible (“smoothness assumption”) and therefore, a particular current density distribution is found. This fundamental assumption of LORETA directly relies on the neurophysiological observation of coherent firing of neighboring cortical neurons during stimulus processing (Pascual-Marqui et al., 1994) and therefore can be seen as a physiologically based constraint. However, this coherent firing has been described on the level of cortical columns, which have a much smaller diameter than the voxels used in the LORETA software; the empirical basis for coherent firing in the millimeter range is not strong enough to fully accept this constraint as a physiological one, even if it might help to produce useful results. While for typical scalp-potentials, coherent firing might be observed in brain volumes as large as or even larger than LORETA voxels. Therefore, the resulting solution is characterized by its relatively low spatial resolution, which is a direct consequence of the smoothness constraint. Taken this together, the solution produces a “blurred-localized” image of a point source, conserving the location of maximal activity, but with a certain degree of dispersion. In the present study, the version of LORETA used is the digitized Talairach atlas available as digitized MRI from the Brain Imaging Centre, Montreal Neurologic Institute, estimating the current source density (microAmperes/mm2) distribution for either single time points or epochs of brain electric activity on a dense grid of 2394 voxels at 7 mm spatial resolution (Pascual-Marqui et al., 1999). The solution space (the three-dimensional space where the inverse EEG problem is solved) was restricted to the gray matter and hippocampus in the Talairach atlas (anatomically based constraint). Localization with regard to spherical and realistic head geometry was done using EEG electrode coordinates reported by Towle et al. (1993).

### Statistical analyses

EEG frequency bands included in the analyses of delta (1.5–6.0 Hz), theta (6.5–8.0 Hz), alpha 1 (8.5–10 Hz), alpha 2 (10.5–12.0 Hz), beta 1 (12.5–18.0 Hz), beta 2 (18.5–21.0 Hz), and beta 3 (21.5–30.0 Hz). For the assessment of treatment effects in OCD patients, a region of interest (ROI) approach was defined as follows: 1. ACC: anterior cingular cortex and 2. OFC: orbito-frontal cortex. These regions were chosen because these have been previously identified with altered activity and response to antidepressant treatment (Fontenelle et al., 2006; Mulert et al., 2007; Schiepek et al., 2007). The first ROI ACC consisted of 25 voxel from the Brodmann areas 24, 25, and 32 and also the Talairach from x = −10 to 11, from y = 3 to 45 and from z = −6 to 8. The second ROI OFC consists of 298 voxel from the Brodmann areas 10, 11, 25, and 47 and also the Talairach from x = −45 to 52, from y = 9 to 65 and from z = −25 to 24.

In order to evaluate group differences between responders and non-responders in regard to age, age of disease onset, gender and data about the psychopathology unpaired *t*-test for independent samples was used. Spearman's correlation coefficients were calculated to relate the measured LORETA current density changes in the defined ROIs vs. the reduction of Y-BOCS scores. In addition, linear as well as robust regression techniques were used to assess the association between density changes and the reduction of Y-BOCS. Huber type M-estimation (Huber and Ronchetti, 1981) as implemented in the R package MASS (Venables and Ripley, 2002; function rlm) was used to down-weight observations with extreme density changes. As further robust technique, which also takes outliers in Y-BOCS scores and leverage points into account, we used MM-estimation (Yohai et al., 1991; Marazzi, 1993; function rlm) which combines M-estimation with the resistance of high breakdown estimators. Approximate *p*-values based on the assumption that the t statistics [estimate/SE(estimate)] are approximately normally distributed were obtained for M-estimators and MM-estimators. Given the exploratory character of the study, statistical significance levels were set to *p* < 0.05 and *p* < 0.10 (statistical trend) and not additionally corrected for multiple comparisons. Statistical analyses were performed using the SPSS software (IBM SPSS, Version 23.0), the statistical software R (version 3.0.1; Team, 2013) or with the implemented LORETA analysis tool that includes a correction for multiple comparisons and does not require any assumption of Gaussianity (Diener et al., 2010). The localization of the differences in activity between the groups of responders and non-responders was assessed by voxel-by-voxel non-paired *t*-test of the LORETA images, based on the power of estimated electric current density, which results in *t* statistic three dimensional images (Mientus et al., 2002). In these images, cortical voxels of statistically significant differences were identified by a non-parametric approach (maximum t-statistic) using randomization strategy that determined the critical probability threshold values for actually observed statistic with corrections for multiple testing (Holmes et al., 1996).

## Results

### All OCD patients compared at baseline and follow-up

#### Descriptive clinical data

Sociodemographic data was compared between the groups of responders and non-responders and no statistical differences were identified. Data concerning the psychopathology of the patients showed a significant reduction over the course of the therapy in all rating scales. After 10 weeks of treatment, 20 of 41 patients fulfilled the criteria for treatment response, defined as a decrease in the Y-BOCS score by at least 50%, see also Tables 1–3 for detailed results.

**Table 1:** **Descriptive socio-demographic data of responders and non-responders**.

**Comparison of responders (*****n*** = 20**) and non-responders (*****n*** = 21**)**			
	**Responder**	**Non-responder**	**Significance**
Age in years	35.0 (±8.8)	34.0 (±10.7)	*p* = 0.758 *t* = 0.311
Age of disease onset	22.3 (±10.5)	21.2 (±8.3)	*p* = 0.716 *t* = 0.367
Duration of illness	12.7 (±9.7)	12.8 (±9.0)	*p* = 0.963 *t* = 0.047
Dose of sertraline (mg)	63.2 (±36.7)	61.9 (±21.8)	*p* = 0.334 χ² = 2.191
Smoking status	5 smoker; 15 non-smoker	5 smoker; 16 non-smoker	*p* = 0.929 χ² = 0.008
Gender	9 female; 11 male	9 female; 12 male	*p* = 0.890 χ² = 0.190

**Table 2:** **Descriptive psychopathology of the patients at baseline and follow-up**.

**Patients with OCD (*****n*** = 41**)**				
	**Baseline mean (*****sd*****)**	**After 10 weeks treatment mean (*****sd*****)**	**Delta (sd)**	**Significance**
Y-BOCS total score	25.29 (±5.78)	14.44 (±7.94)	10.85 (±7.96)	*p* = 0.000 *t* = 8.74
Y-BOCS sub score (compulsions)	11.83 (±3.79)	6.73 (±4.12)	5.10 (±4.33)	*p* = 0.000 *t* = 7.54
Y-BOCS sub score (obsessive thoughts)	13.46 (±2.77)	7.71 (±4.22)	5.76 (±4.43)	*p* = 0.000 *t* = 8.32
HAMD-17	12.78 (±6.08)	9.15 (±7.09)	3.59 (±6.89)	*p* = 0.002 *t* = 3.26
BDI	17.27 (±8.67)	13.94 (±12.70)	4.18 (±10.06)	*p* = 0.002 *t* = 2.42
MOCI	13.20 (±4.16)	8.80 (±5.56)	4.40 (±4.50)	*p* = 0.000 *t* = 5.36

**Table 3:** **Differences of responders and non-responders at baseline and follow-up**.

**Comparison of responders (*****n*** = 20**) and non-responders (*****n*** = 21**)**				
	**Responder**	**Non-responder**	
**BASELINE**			
Y-BOCS total score	25.15 (±6.21)	25.43 (±5.49)	*p* = 0.880 *t* = 0.15
Y-BOCS compulsions	11.80 (±4.20)	11.86 (±3.47)	*p* = 0.962 *t* = 0.05
Y-BOCS obsessive thoughts	13.35 (±2.96)	13.57 (±2.64)	*p* = 0.802 *t* = 0.25
HAMD-17	13.90 (±6.55)	11.65 (±5.53)	*p* = 0.248 *t* = 1.18
MOCI	12.61 (±4.63)	13.83 (±3.92)	*p* = 0.399 *t* = 0.86
BDI	15.21 (±8.31)	19.44 (±8.73)	*p* = 0.140 *t* = 1.51
**AFTER 10 WEEKS TREATMENT**			
Y-BOCS total score	8.30 (±4.04)	20.29 (±6.09)	*p* = 0.000 *t* = 7.39
Y-BOCS compulsions	3.90 (±2.17)	9.43 (±3.71)	*p* = 0.000 *t* = 5.86
Y-BOCS obsessive thoughts	4.40 (±2.44)	10.86 (±2.94)	*p* = 0.000 *t* = 7.64
HAMD-17	8.30 (±7.40)	10.00 (±6.85)	*p* = 0.456 *t* = 0.75
MOCI	7.06 (±5.18)	11.18 (±6.17)	*p* = 0.043 *t* = 2.11
BDI	10.11 (±12.37)	17.78 (±12.17)	*p* = 0.069 *t* = 1.88
**DIFFERENCE BASELINE—WEEK 10**			
Y-BOCS total score	16.85 (±5.82)	5.14 (±4.91)	*p* = 0.000 *t* = 6.97
Y-BOCS compulsions	7.90 (±3.60)	2.43 (±3.14)	*p* = 0.000 *t* = 5.20
Y-BOCS obsessive thoughts	8.95 (±3.52)	2.71 (±2.76)	*p* = 0.000 *t* = 6.33
HAMD-17	5.60 (±5.69)	1.47 (±7.53)	*p* = 0.063 *t* = 1.92
MOCI	5.93 (±5.42)	2.87 (±2.75)	*p* = 0.064 *t* = 1.96
BDI	5.71 (±11.37)	2.65 (±8.62)	*p* = 0.384 *t* = 0.88

#### EEG data

**Figure 1:**
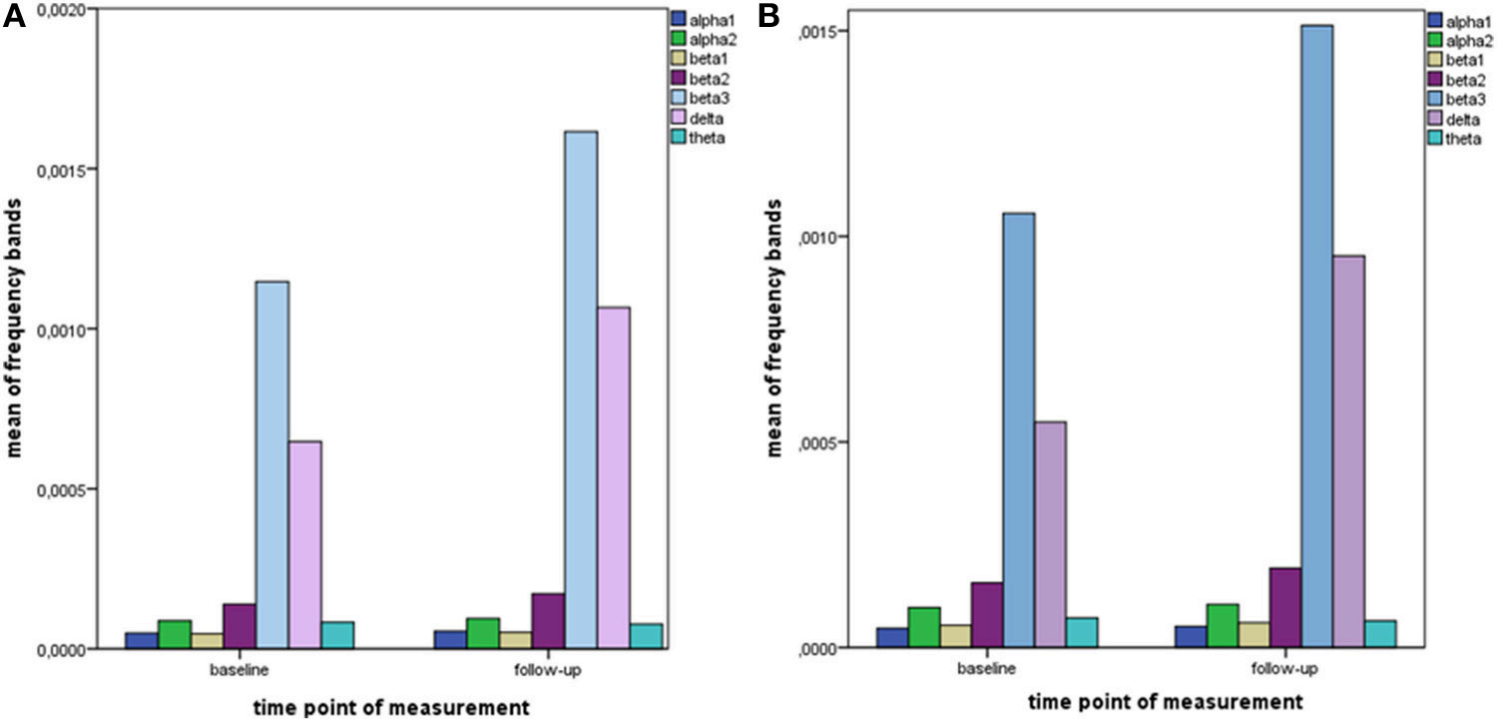
**Descriptive data of EEG frequency bands**. Mean values of all measured EEG frequency bands (in μAmpere/mm^2^) are shown at baseline and at follow-up. **(A)** brain region ACC; **(B)** brain region OFC.

The distribution of EEG frequency bands is shown in Tables 4–7 and Figure 1 for baseline and follow-up and also the comparison of the responders and non-responders (OFC and ACC region) are displayed.

**Table 4:** **Frequency bands of the ACC**.

**Frequency bands (in 10**^**−3**^ μ**V**^**2**^**)**		**alpha1 T0**	**alpha1 T1**	**alpha2 T0**	**alpha2 T1**	**beta1 T0**	**beta1 T1**	**beta2 T0**	**beta2 T1**	**beta3 T0**	**beta3 T1**	**delta T0**	**delta T1**	**theta T0**	**theta T1**
Non-responder	Mean	0.049	0.043	0.107	0.099	0.060	0.061	0.177	0.220	1.306	1.218	0.738	0.526	0.079	0.068
	*n*	21	21	21	21	21	21	21	21	21	21	21	21	21	21
	*sd*	0.0412	0.0363	0.0755	0.0921	0.0522	0.0769	0.1481	0.3323	0.8993	1.0182	0.6295	0.3232	0.0664	0.0574
	Minimum	0.0	0.0	0.0	0.0	0.0	0.0	0.0	0.0	0.4	0.3	0.2	0.2	0.0	0.0
	Maximum	0.2	0.1	0.3	0.4	0.2	0.3	0.6	1.6	3.5	4.9	2.6	1.4	0.2	0.2
Responder	Mean	0.049	0.068	0.067	0.091	0.034	0.042	0.101	0.122	0.980	2.032	0.552	1.633	0.088	0.087
	*n*	20	20	20	20	20	20	20	20	20	20	20	20	20	20
	*sd*	0.0543	0.0816	0.0459	0.0599	0.0217	0.0248	0.0840	0.0765	0.6675	3.5464	0.4261	3.2201	0.0908	0.0716
	Minimum	0.0	0.0	0.0	0.0	0.0	0.0	0.0	0.0	0.2	0.3	0.1	0.2	0.0	0.0
	Maximum	0.2	0.3	0.2	0.3	0.1	0.1	0.4	0.3	3.0	16.0	1.6	014.5	0.4	0.3
All patients	Mean	0.049	0.055	0.087	0.095	0.047	0.051	0.140	0.172	1.147	1.615	0.647	1.066	0.083	0.077
	*n*	41	41	41	41	41	41	41	41	41	41	41	41	41	41
	*sd*	0.0474	0.0631	0.0653	0.0773	0.0419	0.0579	0.1257	0.2460	0.8020	2.5810	0.5415	2.3002	0.0783	0.0647
	Minimum	0.0	0.0	0.0	0.0	0.0	0.0	0.0	0.0	0.2	0.3	0.1	0.2	0.0	0.0
	Maximum	0.2	0.3	0.3	0.4	0.2	0.3	0.6	1.6	3.5	16.0	2.6	0.4.5	0.4	0.3

*Descriptive data about the frequency bands of the ACC region. T0 are baseline values and T1 are values after 10 weeks of treatment in μV^2^. n, number of patients; sd, standard deviation*.

**Table 5:** **Frequency bands of the OFC**.

**Frequency bands (in 10**^**−3**^ μ**V**^**2**^**)**		**alpha1 T0**	**alpha1 T1**	**alpha2 T0**	**alpha2 T1**	**beta1 T0**	**beta1 T1**	**beta2 T0**	**beta2 T1**	**beta3 T0**	**beta3 T1**	**delta T0**	**delta T1**	**theta T0**	**theta T1**
Non-responder	Mean	0.052	0.044	0.130	0.111	0.074	0.073	0.214	0.243	1.275	1073	0.641	0.476	0.076	0.057
	*n*	21	21	21	21	21	21	21	21	21	21	21	21	21	21
	*sd*	0.0510	0.0464	0.1035	0.1073	0.0725	0.1059	0.2016	0.3661	1.1111	0.9726	0.8012	0.3530	0.0787	0.0518
	Minimum	0.0	0.0	0.0	0.0	0.0	0.0	0.0	0.0	0.4	0.2	0.1	0.1	0.0	0.0
	Maximum	0.2	0.2	0.4	0.4	0.3	0.5	0.9	1.7	5.1	4.7	3.9	1.4	0.3	0.2
Responder	Mean	0.042	0.059	0.062	0.099	0.033	0.047	0.098	0.140	0.826	1.974	0.452	1.453	0.068	0.073
	*n*	20	20	20	20	20	20	20	20	20	20	20	20	20	20
	*sd*	0.0453	0.0576	0.0406	0.0737	0.0204	0.0317	0.0765	0.0999	0.5289	3.3293	0.3307	3.0294	0.0672	0.0588
	Minimum	0.0	0.0	0.0	0.0	0.0	0.0	0.0	0.0	0.3	0.4	0.2	0.2	0.0	0.0
	Maximum	0.2	0.2	0.2	0.3	0.1	0.1	0.3	0.4	2.4	14.3	1.3	12.9	0.3	0.2
All patients	Mean	0.047	0.051	0.097	0.105	0.054	0.060	0.157	0.193	1.056	1.513	0.549	0.953	0.072	0.065
	*n*	41	41	41	41	41	41	41	41	41	41	41	41	41	41
	*sd*	0.0480	0.0520	0.0856	0.0915	0.0571	0.0792	0.1631	0.2728	0.8955	2.4384	0.6182	2.1600	0.0725	0.0553
	Minimum	0.0	0.0	0.0	0.0	0.0	0.0	0.0	0.0	0.3	0.2	0.1	0.1	0.0	0.0
	Maximum	0.2	0.2	0.4	0.4	0.3	0.5	0.9	1.7	5.1	14.3	3.9	12.9	0.3	0.2

*Descriptive data about the frequency bands of the OFC region. T0 are baseline values and T1 are values after 10 weeks of treatment in μV^2^. n, number of patients; sd, standard deviation*.

**Table 6:** **Changes of the frequency bands of the ACC**.

**Changes in the frequency bands (in 10**^**−3**^ μ**V**^**2**^**)**		**alpha1 T1–T0**	**alpha 2 T1–T0**	**beta 1 T1–T0**	**beta 2 T1–T0**	**beta 3 T1–T0**	**delta T1–T0**	**theta T1–T0**
Non-responder	Mean	−0.006	−0.064	0.001	0.044	−0.087	−0.211	0.000
	*n*	21	21	21	21	21	21	21
	*sd*	0.0270	0.0624	0.0581	0.2835	0.9188	0.7525	0.0400
	Minimum	−0.1	−0.2	−0.1	−0.2	−2.3	−2.0	0.0
	Maximum	0.1	0.0	0.2	1.2	2.8	1.0	0.0
Responder	Mean	0.019	0.001	0.007	0.021	1.052	1.080	0.000
	*n*	20	20	20	20	20	20	20
	*sd*	0.0475	0.0688	0.0280	0.1024	3.3387	3.2735	0.0800
	Minimum	−0.1	−0.1	0.0	−0.2	−0.2	−0.7	0.0
	Maximum	0.2	0.2	0.1	0.2	0.2	14.2	0.0
All patients	Mean	0.006	−0.032	0.004	0.033	0.469	0.419	0.000
	*n*	41	41	41	41	41	41	41
	*sd*	0.0399	0.0725	0.0455	0.2128	2.4596	2.4086	0.06
	Minimum	−0.1	−0.2	−0.1	−0.2	−2.3	−2.0	0.0
	Maximum	0.2	0.2	0.2	1.2	14.0	14.2	0.0

**Table 7:** **Changes of the frequency bands of the OFC**.

**Changes in the frequency bands (in 10**^**−3**^ μ**V**^**2**^**)**		**alpha1 T1–T0**	**alpha 2 T1–T0**	**beta 1 T1–T0**	**beta 2 T1–T0**	**beta 3 T1–T0**	**delta T1–T0**	**theta T1–T0**
Non-responder	Mean	–0.007	–0.019	0.001	0.000	–0.202	–0.165	–0.019
	*n*	21	21	21	21	21	21	21
	*sd*	0.0450	0.0928	0.0965	0.3400	1.1531	0.7782	0.0724
	Minimum	–0.1	–0.2	–0.1	0.0	–3.9	–3.3	–0.3
	Maximum	0.1	0.3	0.4	0.0	2.9	0.7	0.1
Responder	Mean	0.017	0.037	0.013	0.000	1.148	1.001	0.005
	*n*	20	20	20	20	20	20	20
	*sd*	0.0393	0.0776	0.0342	0.1200	3.1715	3.0062	0.0626
	Minimum	0.0	–0.1	0.0	0.0	–1.4	–0.7	–0.2
	Maximum	0.1	0.2	0.1	0.0	12.5	12.2	0.1
All patients	Mean	0.005	0.008	0.006	0.000	0.456	0.404	0.007
	*n*	41	41	41	41	41	41	41
	*sd*	0.0436	0.0893	0.0726	2.4309	2.4309	2.2235	0.0681
	Minimum	–0.1	–0.2	–0.1	0.0	–3.9	–3.3	–0.3
	Maximum	0.1	0.3	0.4	0.0	12.5	12.2	0.1

### Responders compared at baseline and follow-up

**Figure 2:**
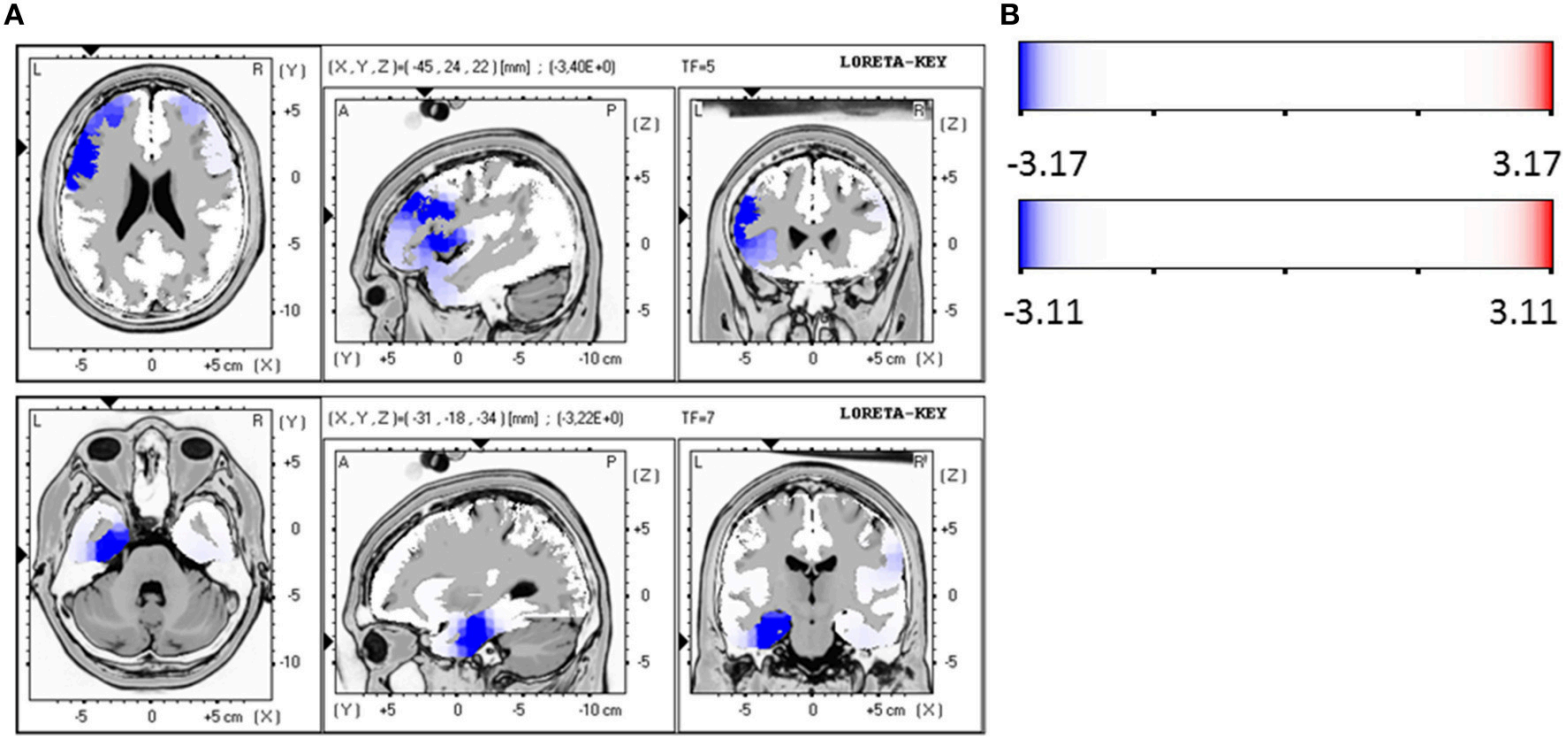
**(A)** LORETA images for responders at baseline and follow-up: Images on top show voxel-wise current density differences in the frequency band beta 1 for responders at baseline compared to follow-up. In the bottom line the same is shown for the frequency band beta 3. The blue color indicates a statistical significant effect (images in the top line: *t*_max_ = −3.40, xyz = −45, 24, 22, *p* < 0.05 and bottom line: *t*_max_ = −3.22, xyz = −31, −18, −34) of reduced current density in the MNI-MRI-standard space. **(B)** Corresponding color bar.

When the time points at baseline and follow-up were compared, responders exhibited significantly lower brain electrical resting activity in the LORETA analyses in the beta 1 (12.5–18.0 Hz; *t* = 3.17. *p* < 0.05) and beta 3 (21.5–30.0 Hz; *t* = 3.11. *p* < 0.05) frequency band, especially in the pre- and postcentral gyri of the frontal and parietal lobe. This indicates an increase in activity over the course of treatment. Results are shown in Table 8 and Figures 2A,B.

**Table 8:** **Characteristics of LORETA current density differences for responders at baseline and follow-up**.

**Frequency band**	**Talairach coordinates X Y Z**			***t*-value (significance)**	**Anatomy**
beta 1	−45	24	22	3.40 (*p* < 0.05)	BA 46 (medial frontal gyrus)
	−52	24	29	3.40 (*p* < 0.05)	BA 46 (medial frontal gyrus)
	−45	24	29	3.40 (*p* < 0.05)	BA 46 (medial frontal gyrus)
	−24	45	29	3.18 (*p* < 0.05)	BA 10 (superior frontal gyrus)
	−31	52	29	3.18 (*p* < 0.05)	BA 10 (superior frontal gyrus)
beta 3	−31	−18	−34	3.22 (*p* < 0.05)	BA 20 (uncus. Limbic cortices)
	−31	−11	−27	3.22 (*p* < 0.05)	BA 20 (uncus. Limbic cortices)
	−31	−18	−27	3.22 (*p* < 0.05)	BA 36 (parahippocampal gyrus)

*BA, Brodmann area*.

Within the ROI analysis of the brain area OFC responders exhibited a significantly lower brain electrical resting activity in the alpha 2 (10.5–12.0 Hz; *t* = 2.15. *p* < 0.05) frequency band where time points before and after treatment were compared. For the frequency bands alpha 1 (8.5–10.0 Hz; *t* = 2.05. *p* < 0.10) and beta 1 (12.5–18.0 Hz; *t* = 1.80. *p* < 0.10) a trend toward a reduced activity was evaluated. In addition, ROI analysis of the area ACC revealed a trend toward a reduced activity in the frequency band alpha 1 (8.5–10.0 Hz; *t* = 1.77. *p* < 0.10) when baseline was compared to follow-up.

### Non-responders compared at baseline and follow-up

**Figure 3:**
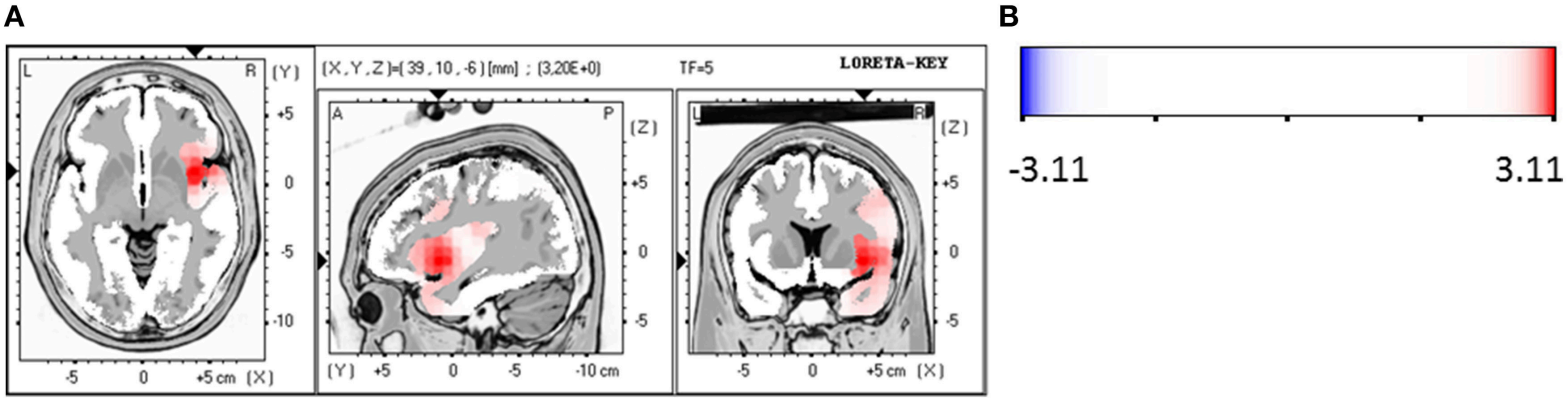
**(A)** LORETA images for non-responders at baseline compared to follow-up: voxel-wise current density differences in the frequency band beta 1 for non-responders. Red areas represent brain regions showing significantly higher activity at baseline compared to follow-up. The red color indicates a statistical significant effect (*t*_max_ = 3.20, xyz = 39, 10, −6 *p* < 0.05) of reduced current density in the MNI-MRI-standard space. **(B)** Corresponding color bar.

For the patient group of the non-responders a significantly higher brain electrical resting activity according to the LORETA analysis could be shown in the beta 1 (12.5–18.0 Hz; *t* = 3.11. *p* < 0.05) frequency band when baseline was compared to follow-up, indicating that brain activity decreased over the course of treatment. A similar trend toward a higher activity in the non-responder group was detected for the frequency bands alpha 1 (8.5–10.0 Hz; *t* = 2.90. *p* < 0.10), alpha 2 (10.5–12.0 Hz; *t* = 2.92. *p* < 0.10) and beta 2 (18.5–21.0 Hz; *t* = 2.73. *p* < 0.10). Over the course of treatment the activity diminished. In the ROI analysis no significant differences were found for the non-responder group. Table 9 shows the significant results and Figures 3A,B displays the current density of the LORETA analysis for beta 1.

**Table 9:** **Characteristics of LORETA current density differences for non-responders**.

**Frequency band**	**Talairach coordinates X Y Z**			***t*-value (significance)**	**Anatomy**
beta 1	39	10	−6	3.20 (*p* < 0.05)	BA 13 (sub-lobar)

*BA, Brodmann area*.

### Responders vs. non-responders at baseline

**Figure 4:**
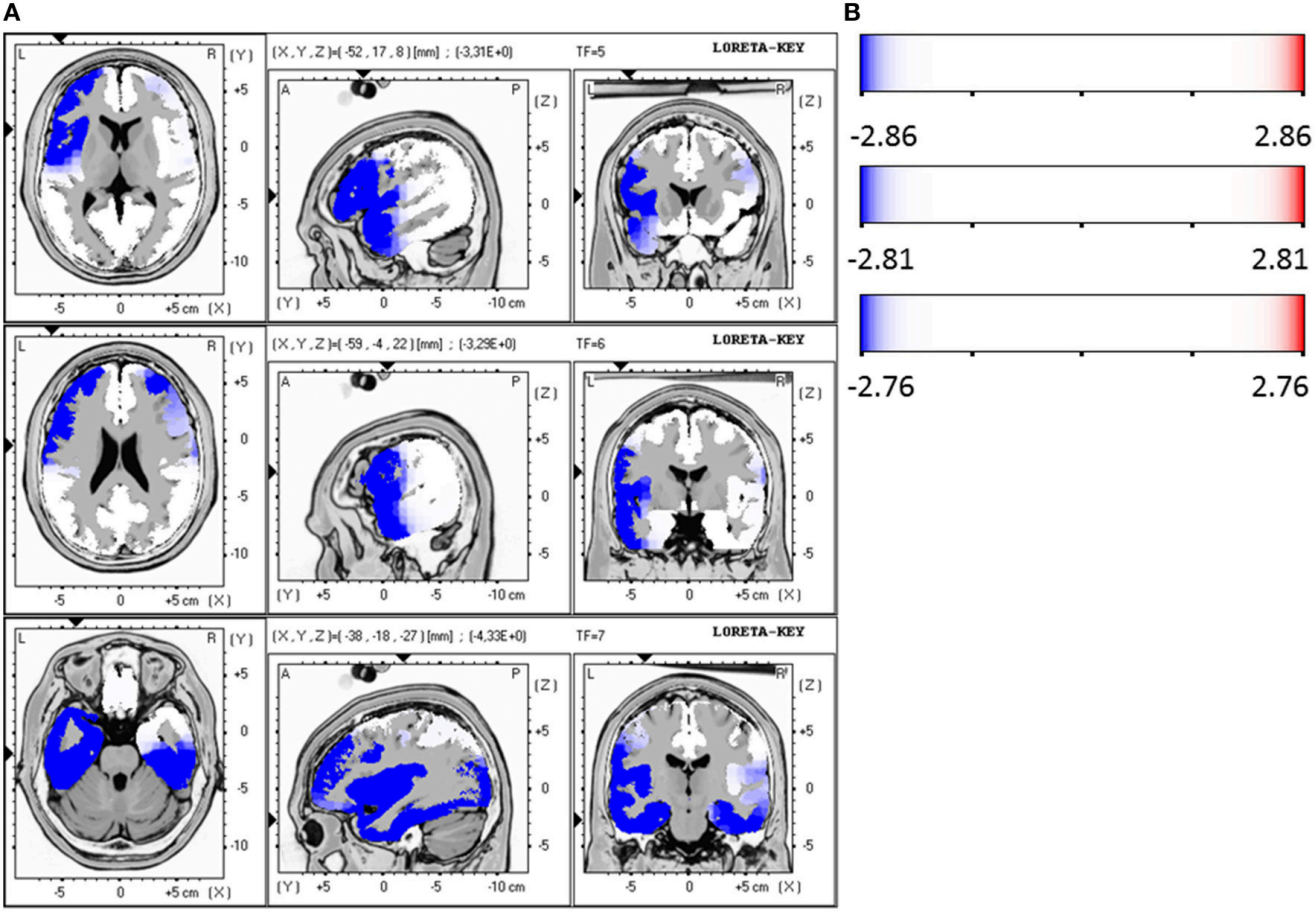
**(A)** LORETA images for responders vs. non-responders at baseline: Images on top show voxel-wise current density differences in the frequency band beta 1 at baseline. The middle line contains the frequency band beta 2, in the bottom line the same is shown for the frequency band beta 3. Blue areas represent brain regions showing significantly lower LORETA values at baseline. The blue color indicates a statistical significant effect (images in the top line: *t*_max_ = −3.31, xyz = −52, 17, 8 *p* < 0.05; middle line: *t*_max_ = −3.29, xyz = −59, −4, 22 and bottom line: *t*_max_ = −4.33, xyz = −38, −18, −27) of reduced current density in the MNI-MRI-standard space. **(B)** Corresponding color bar.

Responders were compared to non-responders before therapy at baseline, where a significant hypoactivity for responders was found in the LORETA analyses in the frequency bands beta 1 (12.5–18.0 Hz; *t* = 2.86, *p* < 0.05), beta 2 (18.5–21.0 Hz; *t* = 2.81, *p* < 0.05) and beta 3 (21.5–30.0 Hz; *t* = 2.76, *p* < 0.05). Precisely, the brain regions that were affected by the hypoactivity were for beta 1 the medial/superior frontal gyrus (12 voxels, BA 9/10, *p* < 0.05, *t*-values between −2.90 and −3.31) and the inferior frontal gyrus, precentral gyrus (5 voxels, BA 44, *p* < 0.05, *t* = −3.31). For beta 2 the medial/superior frontal gyrus (9 voxels, BA 10, *p* < 0.05, t-values between −2.88 and 3.03) and medial/inferior temporal gyrus (4 voxels, BA 20, 21, *p* < 0.05, *t* = 3.01) and precentral gyrus (3 voxels, BA 6, *p* < 0.05, *t* = 3.29). And for beta 3 the medial/superior frontal gyrus (BA 10, *p* < 0.05, 13 voxels, *t*-values between 2.80 and −3.04) and fusiform gyrus (BA 20/37, 21 voxels, *p* < 0.05, *t*-values between −2.97 and −4.06) and parahippocampal gyrus (5 voxels, *p* < 0.05, *t*-values between −2.97 and −4.33) and medial/inferior temporal gyrus (BA 37, 6 voxels, *p* < 0.05, *t*-values between −3.00 and −4.06) were identified. Figures 4A,B summarize the results.

The ROI analysis revealed that the area ACC shows a significantly reduced activity of the responders in comparison to non-responders in the frequency band alpha 2 (10.5–12.0 Hz; *t* = 2.06, *p* < 0.05) at baseline. Additionally, a trend toward a reduced activity was found for beta 1 (12.5–18.0 Hz; *t* = 2.06, *p* = 0.05) and beta 2 (18.5–21.0 Hz; *t* = 2.04, *p* = 0.05). The other investigated area OFC had also a reduced activity for the group of responders compared to non-responders in the frequency bands alpha 2 (10.5–12.0 Hz; *t* = 2.81, *p* < 0.01), beta 1 (12.5–18.0 Hz; *t* = 2.50, *p* < 0.05) and beta 2 (18.5–21.0 Hz; *t* = 2.50, *p* < 0.05) at baseline.

### Responders vs. non-responders at follow-up

The two groups of responders and non-responders did not show significant differences with regard to LORETA and ROI analyses after 10 weeks at follow-up.

### Correlation of the psychopathology with electrophysiological data

The psychopathology of the OCD patients measured with the aid of Y-BOCS was correlated with the brain activity of the ROI areas OFC and ACC at baseline and follow-up. The activity change was defined as: brain activity after 10 weeks of treatment minus activity at baseline. Using Spearman's correlation coefficient a positive correlation between the brain activity change in the area ROI OFC and the reduction of the Y-BOCS scores was identified for the frequency bands alpha 2 (rho = 0.40, *p* = 0.010), beta 3 (rho = 0.42, *p* = 0.006), delta (rho = 0.33, *p* = 0.038), theta (rho = 0.34, *p* = 0.031), alpha 1 (rho = 0.38, *p* = 0.015), and beta1 (rho = 0.34, *p* = 0.028).

**Figure 5:**
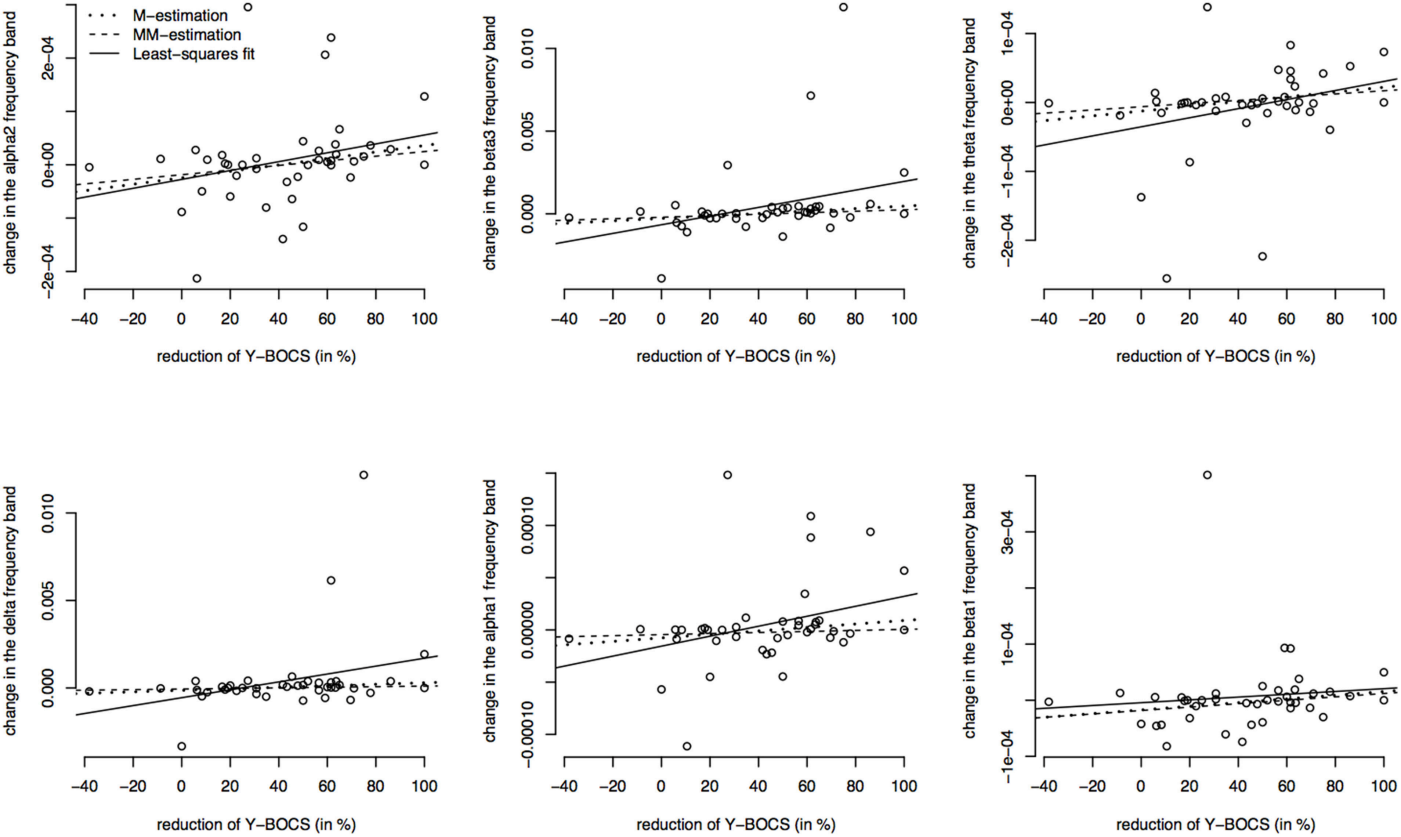
**Correlations between the psychopathology [reductions of Y-BOCS scores (%)] and the change in frequency bands (time point 10 weeks minus baseline) in the OFC**. Frequency band alpha 2, beta3, theta, delta, alpha 1, and beta 1 are shown (in μAmpere/mm^2^) and regression lines obtained through M-estimation, MM-estimation and the least squares method.

Since the data contains many outliers, linear regression was not appropriate and robust regression techniques were applied. Figure 5 shows the regression lines obtained by Huber-type M-estimation (dotted line) and MM-estimation (dashed line). In addition, the regression line of the classical least squares fit is shown (solid line) to assess the influence of outlying observations. For five of six frequency bands the slope of the least squares regression line is larger than that of the robust techniques, indicating that the outlying observations induce a larger correlation between psychopathology and electrophysiological data. The approximate *p*-values for testing the regression coefficients obtained through M-estimation or MM-estimation are very small (Table 10), indicating that there is an association even after taking outliers and influential points into account.

**Table 10:** **Estimated regression coefficients from robust regression (M-estimation and MM-estimation), corresponding ***t***-values and approximate ***p***-values for OFC**.

	**Regression coefficient M-estimation in 10^−7^**	**Regression coefficient MM-estimation in 10^−7^**	***t*-value (*p*-value) M-estimation**	***t*-value (*p*-value) MM-estimation**
alpha 2	6.05	4.33	2.18 (*p* = 0.0357)	1.88 (*p* = 0.0683)
beta 3	74.10	45.00	2.76 (*p* = 0.0088)	1.93 (*p* = 0.0603)
theta	3.46	2.32	2.19 (*p* = 0.0344)	1.73 (*p* = 0.0912)
delta	43.10	16.70	2.18 (*p* = 0.0351)	0.92 (*p* = 0.3607)
alpha 1	1.67	0.505	1.94 (*p* = 0.0592)	0.73 (*p* = 0.4717)
beta 1	3.27	3.04	1.99 (*p* = 0.0539)	1.81 (*p* = 0.0784)

*T-values are computed as t = estimate/SE(estimate) and p-values are obtained using the Wald test.frequency band*.

**Figure 6:**
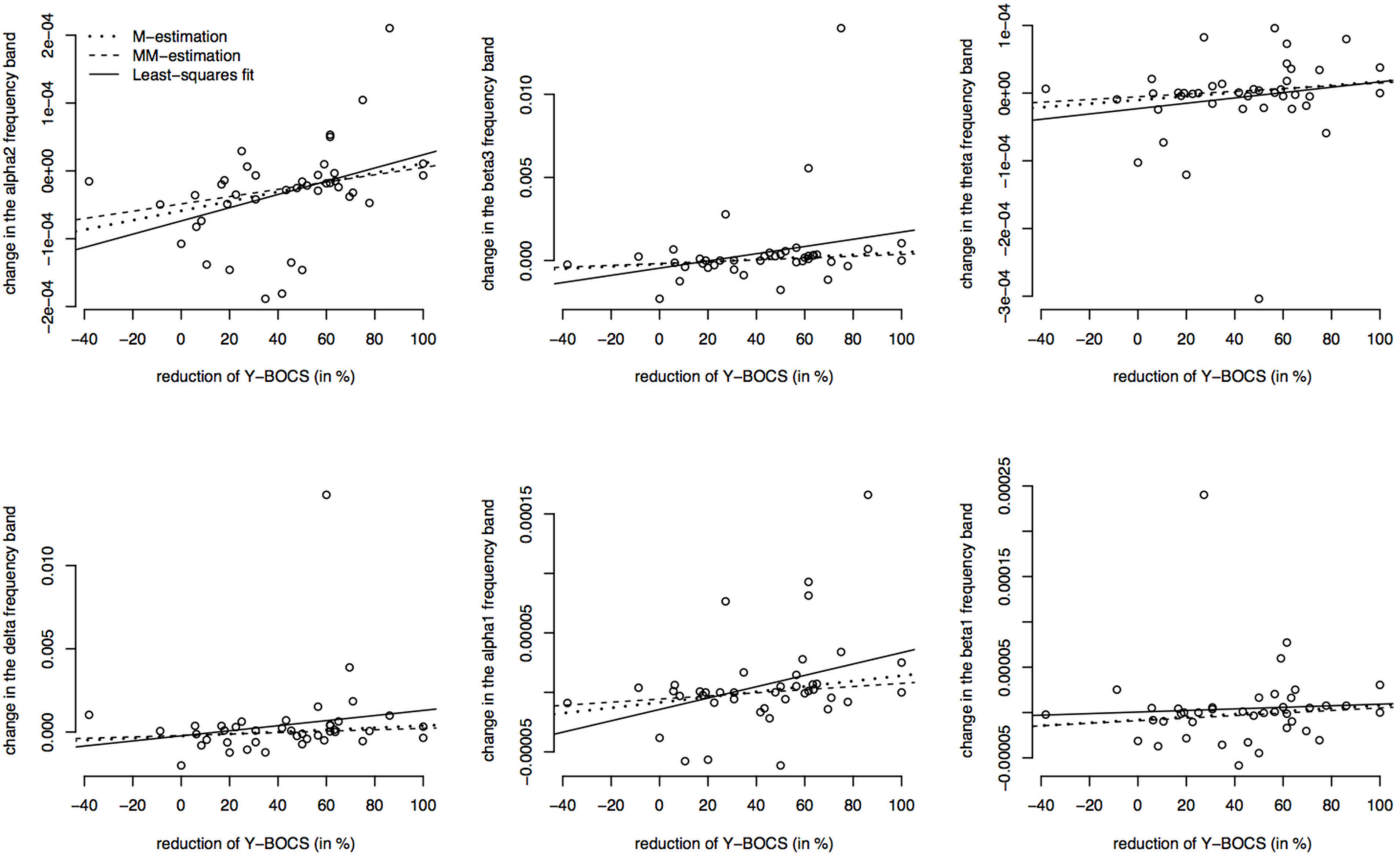
**Correlations between the psychopathology [reduction of Y-BOCS scores (%)] and the change in frequency bands (time point 10 weeks minus baseline) in the ACC**. Frequency bands alpha 2, beta3, theta, delta, alpha 1, and beta 1 are shown (in μAmpere/mm^2^) and regression lines obtained through M-estimation, MM-estimation and the least squares method.

Furthermore, the brain activity of the other region ACC was correlated with the psychopathology of the OCD patients measured with Y-BOCS at baseline and follow-up. Again, the activity change was as explained above, and a positive correlation between the brain activity in the ROI ACC and the reduction of Y-BOCS scores was identified using Spearman's correlation coefficient. The frequency bands delta (rho = 0.33, *p* = 0.035), alpha 1 (rho = 0.36, *p* = 0.019), alpha 2 (rho = 0.34, *p* = 0.031), and beta 3 (rho = 0.38, *p* = 0.015) are shown in Figure 6. Also shown are the regression lines for the least squares fit and the robust regression methods. The findings are very similar to those for the ROI area OFC: outlying observations induce a larger correlation between psychopathology and electrophysiological data since the robust regression methods yield less steep regression lines. Approximate *p*-values are shown in Table 11.

**Table 11:** **Estimated regression coefficients from robust regression (M-estimation and MM-estimation), corresponding ***t***-values and approximate ***p***-values for ACC**.

	**Regression coefficient M-estimation in 10^−7^**	**Regression coefficient MM-estimation in 10^−7^**	***t*-value (*p*-value) M-estimation**	***t*-value (*p*-value) MM-estimation**
alpha 2	6.98	5.37	2.56 (*p* = 0.0144)	2.11 (*p* = 0.0410)
beta 3	70.60	54.80	2.49 (*p* = 0.0173)	2.05 (*p* = 0.0473)
theta	2.72	2.02	1.73 (*p* = 0.0910)	1.25 (*p* = 0.2205)
delta	62.206	42.50	1.48 (*p* = 0.1460)	1.06 (*p* = 0.2945)
alpha 1	2.25	1.32	2.09 (*p* = 0.0435)	1.64 (*p* = 0.1098)
beta 1	1.59	1.40	1.36 (*p* = 0.1812)	1.15 (*p* = 0.2574)

*T-values are computed as t = estimate/SE(estimate) and p-values are obtained using the Wald test.frequency band*.

## Discussion

The present study aimed to identify possible predictors of treatment response in patients suffering from OCD. We found that treatment responders exhibited already at baseline significantly lower brain electrical activity in higher frequency bands compared to non-responders and these findings were confirmed with ROI analyses that also revealed a significantly lower brain electrical resting activity in the ACC of responders. Over a 10 week course of treatment, the responder group had an increase in brain electric activity from baseline to follow-up, confirmed by LORETA analyses showing significantly lower brain electrical activity in the beta frequency bands, the same was true for the ROI analysis of the OFC brain area of responders. In addition, the group of non-responders exhibited the opposite findings: These OCD patients had a significantly higher brain electrical resting activity in the beta frequency band when baseline was compared to follow-up. The presented data clearly indicate that the brain activity increases in responders, whereas in non-responders brain activity decreases over the course of treatment. Therefore, the collection of electromagnetic tomography data might eventually help to distinguish between OCD patients who will respond to treatment and those who do not respond already before the onset of therapy.

Regarding lower electrical activity in responders to OCD treatment our results are consistent with those of previous studies: In particular, one pilot study found that lower pretreatment activity in the beta band within the rostral anterior cingulate and the medial frontal gyrus was associated with a better therapeutic response (Fontenelle et al., 2006). Based on these preliminary data, our group has replicated these findings on a larger OCD patient sample with stricter response criteria: We chose a reduction of at least 50% of the YBOCS score instead of 35%. In addition a standardized treatment with only one antidepressant (sertraline) was determined, and further investigations of electrical resting activity at different time points as well as correlations of the psychopathology with these data were added.

In order to address the question whether psychopathology of OCD was associated with brain activity, OCD symptom severity—assessed with the aid of Y-BOCS—was correlated with ROI analyses. A positive correlation between frequency bands of both brain areas, the ROI OFC and the ROI ACC, with a reduction of Y-BOCS scores was identified. These findings indicate that an improvement of OCD symptoms is associated with an altered brain activity. In a previous study our research group has already identified evidence for symptom-related electrophysiological alterations in unmedicated patients with OCD. It has been shown that patients presenting with high levels of obsessions had higher absolute EEG power measures, especially for the faster frequencies, whereas patients with high compulsion scores were likely to have lower absolute EEG power, especially of slower frequencies (Andreou et al., 2013).

Also, a previous PET study investigated the correlation between symptomatology of OCD and brain activity and showed that in medicated OCD patients, the decrease in right orbitofrontal metabolism was directly correlated with two measures of OCD improvement (Swedo et al., 1992), indicating that brain activity and OCD psychopathology are mutually dependent parameters.

In our study, the investigated brain areas ACC and OFC had already been linked to an abnormal activity in OCD patients and were therefore proposed to play a role in the pathophysiology of the disease. However, other brain regions like the dorsolateral prefontal cortex and the inferior frontal gyrus that have previously shown abnormalities in OCD patients (Goncalves et al., 2015; Tang et al., 2015) have not been investigated in our study. Andreou et al. have examined brain activity distribution with LORETA and reported an increased P300-related activity predominantly in the left OFC, but also in left prefrontal, parietal and temporal areas in OCD patients compared to controls (Andreou et al., 2013). Moreover, especially the beta frequency bands have already been shown to have a lower power also in the frontal brain regions (Kuskowski et al., 1993). For the other investigated region ACC, LORETA analyses revealed an excess current source density in the beta frequencies in the cingulate gyrus in OCD patients compared to controls (Sherlin and Congedo, 2005). Also fMRI studies strengthen the evidence for ACC dysfunction in OCD (Brennan et al., 2015). In addition, another LORETA study in OCD patients has shown that there are differences in the frequency bands between patients and controls. The authors found that OCD patients had an increased current density for beta in the frontal, parietal and limbic lobes (Velikova et al., 2010). However, our data are difficult to compare to studies investigating OCD patients in comparison to healthy controls, since we looked only at OCD patients. Still, our findings that patients who are going to be treatment responders have lower activation levels in relevant brain areas may point toward a rather normal brain activity level, possibly making them more likely to respond to treatment.

The present study has several strengths and limitations. Regarding its strength our study has a comparatively large patient sample with a homogenous standardized treatment for all OCD patients. With the criteria of response chosen at the level of at least 50% reduction in the Y-BOCS, these rather strict response criteria identified only responders who really had a clinically significant improvement of their OCD symptoms. In terms of limitations, all our patients were only unmedicated and not drug naïve. Furthermore, in this study no testing for multiple comparisons was done due to the exploratory character of the study. Therefore, based on the findings by Fontenelle et al. (2006) our results can only be seen as the next step toward the clinical applicability of predictive electromagnetical markers in OCD patients. However, the final clinical implementation of EEG markers in OCD cannot yet be reached with the present results.

Some authors have suggested that an increase in brain activity is due to an improvement of depressive symptoms (Kennedy et al., 2001). However, our sample of OCD patients did not distinguish between groups of responders and non-responders with regard to depressive symptoms. Study participants had a mean HAMD-17 score of 12.78 indicating only mild depressive symptoms (cut-off HAMD-17; Zimmerman et al., 2013) before treatment, and a score of 9.15 after treatment. Responders and non-responders did not show a significant difference regarding HAMD-17 or BDI scores for depressive symptoms. Therefore, a contamination of our results caused by the influence of depressive symptoms seems unlikely in our investigated sample.

It would also be of interest to link our data to research on brain connectivity in order to broaden the picture of OCD psychopathology. Consistent with neurobiological models of OCD, OFC, and basal ganglia have been identified to be hyperconnected in unmedicated patients, and antidepressant medication may reduce connectivity within corticobasal ganglia-thalamo-cortical circuits in OCD (Beucke et al., 2013). Also, an altered global brain connectivity in dorsal and ventral striatum of OCD patients has been shown, as well as complex disturbances in PFC networks which could contribute to disrupted cortical-striatal-cerebellar circuits in OCD (Anticevic et al., 2014).

Altogether, our results suggest that measuring brain activity with LORETA could eventually be an efficient and simple technique in order to identify OCD patients likely to respond to treatment. We hope that future studies will continue to rigorously examine whether the biomarkers we have shown in the present study might qualify as an effective predictor of treatment response in OCD patients. More generally, our findings illustrate the potential utility of resting EEG and LORETA analyses for identifying biomarkers of treatment response, thereby facilitating a personalized clinical approach to treating patients suffering from OCD.

### Conflict of interest statement

The authors declare that the research was conducted in the absence of any commercial or financial relationships that could be construed as a potential conflict of interest.
